# The effect of verdict system on juror decisions: a quantitative meta-analysis

**DOI:** 10.1080/13218719.2023.2272912

**Published:** 2024-01-11

**Authors:** Elaine Jackson, Lee Curley, Fiona Leverick, Martin Lages

**Affiliations:** a School of Psychology and Neuroscience, University of Glasgow, Glasgow, UK; b School of Psychology, The Open University, Milton Keynes, UK; c School of Law, University of Glasgow, Glasgow, UK

**Keywords:** cognitive bias, conviction rate, juror decisions, logistic regression, not-proven verdict, random effects, three-verdict system, two-verdict system

## Abstract

We study the effect of the Scottish three-verdict system (guilty, not guilty, not proven) and the Anglo-American two-verdict system (guilty, not guilty) on juror decisions by combining data sets from 10 mock trials reported in suitable studies. A logistic regression with random effects uses the exact number of convictions and acquittals in 10 mock trials from a total of 1778 jurors to reliably estimate the effect of verdict system. We found a statistically significant verdict effect suggesting that the odds for a conviction by a juror are about 0.6 times or 40% lower under the three-verdict system than under a conventional two-verdict system. Possible explanations and implications of this verdict effect are discussed. This finding helps to better understand juror decision making in the context of the current reform of the Scottish three-verdict system into a two-verdict system.

## Introduction

Scotland has a separate and distinct legal system from the rest of the UK. One of the key differences is in respect of its criminal verdict system. Like many other legal systems built on the Anglo-American model, it uses juries to determine guilt in criminal cases. But rather than the two-verdict system (*guilty* and *not guilty*), utilised by the majority of Anglo-American countries, the Scottish system (*guilty*, *not guilty* and *not proven*) offers jurors the choice of three verdict options (Chalmers et al., 2021a, 2022). The *not proven* verdict operates as a second acquittal verdict, with no legal definition, and judges are dissuaded from trying to define the verdict to the jury (Curley et al., 2022). Legally the *not proven* verdict has exactly the same effect as the *not guilty* verdict, and there is no distinction in law between the two verdicts of acquittal, with both verdicts being given when the prosecution has failed to prove their case beyond reasonable doubt. Jurors are simply told that they have two alternative options with the same consequences (Judicial Institute for Scotland, 2022). Furthermore, the Scottish jury system uses juries with 15 members (rather than 12), and it reaches a jury verdict via a simple majority rule, rather than requiring (almost) unanimity amongst jurors (Chalmers et al., 2020).

In recent years, there has been increased scrutiny of the *not proven* verdict, particularly by charitable organisations and activists (Rape Crisis Scotland, 2021) who claim that the verdict is confusing and that it leads to a disproportionate number of acquittals in sexual violence trials. In 2022, the Scottish Government announced that it would introduce legislation to end the use of the *not proven* verdict (Scottish Government, 2022). This comes at a time when there is increased interest in the *not proven* verdict elsewhere, with proponents of the Scottish verdict system arguing that *not proven* – or something similar – should be introduced into legal systems that operate without it (e.g. Allan, 2017; Phalen, 2018; Picinali, 2022). One of the key difficulties in assessing the case for the *not proven* verdict is that until recently, there had been relatively few empirical studies that have investigated the effect of verdict system on juror decision making.

Now, however, a sufficient number of experimental studies comparing the two verdict systems is available. The aim of the current study is therefore to conduct a quantitative meta-analysis on the results from carefully selected studies that investigated the differential effect of verdict systems on juror decisions – specifically, the impact of the third verdict option of *not proven* on juror preferences for conviction. Although there is evidence from individual studies that the Scottish verdict system reduces convictions, other studies suggest that the availability of the *not proven* verdict does not significantly affect conviction rates. This provides a compelling rationale for undertaking a meta-analysis that addresses controversies arising from conflicting or ambiguous claims.

### Background

The *not proven* verdict has a long history in the Scottish legal system. The original verdicts in Scottish criminal cases were essentially *guilty* and *not guilty*. However, there was a lack of consistency in what they were called, with guilt being declared through terms such as ‘had done wrangis’ (scots) or ‘convictus’ (latin), and innocence being delivered through names such as ‘made qwyt’ or ‘clene and sakles’ (scots; Barbato, 2004). In the early seventeenth century, a change in procedure meant that jurors did not give general verdicts on criminal cases, meaning they no longer made decisions in relation to the guilt of the accused. Instead, they were asked to declare whether various facts were proven or not proven (in other words, they gave special verdicts), and then the judge would declare the accused innocent or guilty on the basis of these factual findings. This procedure eventually disappeared, and juries once again became the body that pronounced a general, or overall, verdict on the case. But the terminology of *not proven* remained, and juries started to use this as a general verdict, alongside *guilty* and *not guilty* (Chalmers et al., 2021a; Curley et al., 2022; Willock, 1966).

Although Scotland is unusual in having a differentiated verdict system, it is not unique. In Israel, there are also two acquittal verdicts – a full acquittal and a ‘doubt-based acquittal’, in which doubt exists regarding the acquitted person’s innocence (Vaki & Rabin, 2021). There was also a case where a Washington state judge gave a *not proven* over a *not guilty* verdict (Bray, 2005). Until a major reform of the Code of Criminal Procedure for Italy in 1988, the Italian criminal justice system differentiated between full acquittals and acquittals ‘for lack of evidence’ (Gebbie et al., 1999; Picinali, 2022).

There has been much debate over the merits of the *not proven* verdict in the Scottish verdict system. Its proponents argue that it is a safeguard against wrongful conviction in borderline cases that do not quite reach the legal threshold of proof beyond reasonable doubt (Allan, 2017; Phalen, 2018). In such cases, jurors can opt for the *not proven* verdict, whereas faced with an identical case in a two-verdict system, they may be tempted to convict (Curley et al., 2022). Opponents of the *not proven* verdict argue that it leaves an unjust stigma on an acquitted person (Hope et al., 2008) and allows jurors and juries to use it as a compromise verdict in difficult cases, rather than fully discussing the evidence (Chalmers et al., 2022). Debate in recent times has also focused on sexual offence cases, with a campaign to abolish the *not proven* verdict being conducted by Rape Crisis Scotland, on the basis that it causes unnecessary distress to those whose allegations of sexual assault conclude with a *not proven* verdict being returned (Chalmers et al., 2022).

For a long time, empirical studies of the effect of the *not proven* verdict were few and far between. However, several research teams have empirically investigated whether the *not proven* verdict, as a third option, has an effect on juror decision making. Smithson et al. (2007) report that the *not proven* verdict significantly reduced the frequency of *not guilty* verdicts; however, this had little effect on the frequency of *guilty* verdicts returned. Hope et al. (2008) support this finding and suggest in two separate studies that the presence of the *not proven* verdict reduced the frequency of *not guilty* verdicts but did not reduce the frequency of *guilty* verdicts (save in one experimental condition where the evidence against the accused was moderately strong). Similarly, Curley et al. (2019) report that jurors assigned to the three-verdict system returned significantly lower levels of *not guilty* verdicts than those assigned to the two-verdict system whereas convictions were not significantly different between verdict systems.

Ormston et al. (2019) conducted a large-scale multifactorial experiment, investigating in a between-subjects counter-balanced design firstly, the effect of verdict system (three vs. two), secondly, the effect of jury size (15 vs. 12) and the majority rule (simple vs. unanimous) on (pre- and post-deliberation) juror and jury verdicts in a physical assault and rape mock trial. For the purposes of the current analyses, only the effect of verdict systems on pre-deliberation juror decisions in corresponding experimental conditions are considered, because the remaining two factors only influenced post-deliberation verdicts. Ormston et al. (2019) report that the proportion of *guilty* juror verdicts was reduced for the three-verdict system compared to the two-verdict system. However, this was only statistically significant for the physical assault mock trial but not for the rape mock trial. When differentiating between the two acquittal verdicts, it was found that jurors preferred the *not proven* option to that of the *not guilty* option.

Finally, the study by Curley et al. (2022) suggests that jurors returned significantly fewer *guilty* verdicts within the three-verdict system than within the two-verdict system. Interestingly, a test of an experimental verdict system (with *proven* and *not proven* verdict) indicated that jurors returned significantly fewer convictions than the two-verdict English system, whereas the number of convictions in this *proven* – *not proven* verdict system was comparable to that in the current Scottish system.

It seems likely that the studies by Ormston et al. (2019) and Curley et al. (2022) give more accurate results because in these studies the stimulus material was more realistic than in the earlier studies by Curley et al. (2019), Hope et al. (2008) and Smithson et al. (2007). The studies by Ormston et al. (2019) and Curley et al. (2022) are also more ecologically valid according to the six criteria outlined by Willmott et al. (2021), with the former study meeting five of the six criteria and the latter study meeting four. They do not meet all criteria because Ormston et al. (2019) and Curley et al. (2022) did not collect participants from the electoral roll, and Curley et al. (2022) also omitted deliberations due to the Covid-19 pandemic. However, individual studies, no matter how ecologically valid they are, can only provide limited evidence, whereas a quantitative meta-analysis on conviction rates can establish a more precise and robust summary estimate of the verdict effect.

There are a number of possible reasons as to why the availability of the *not proven* verdict may influence the frequency by which *guilty* verdicts are given. Firstly, the availability of the *not proven* verdict may polarise the other available options (*guilty* and *not guilty*), leading jurors to use the *not proven* verdict as a compromise (Chalmers et al., 2022; Curley et al., 2021; Hope et al., 2008). Secondly, there may be differences between legal meaning and lay semantic interpretation of the *not guilty* and *not proven* verdicts – namely, as a distinction between ‘truth’ and ‘proof’ (Jackson, 1998). Terms such as ‘proven’ may encourage jurors to focus on the negatives or weaknesses of the Crown’s (prosecution) evidence since the burden of proof lies with them to demonstrate the guilt of the accused beyond reasonable doubt, thus leading jurors to be more likely to acquit (Curley et al., 2021, 2022; Hope et al., 2008; Jackson, 1998; McKenzie, 1985). Finally, introducing a third option may be related to the so-called ‘decoy effect’ in preference and consumer behaviour (Huber et al., 1982; Kahneman & Tversky, 1984). Although plausible, it remains unknown whether passing a verdict invokes similar decision heuristics as preferential choices (Hope et al., 2008).

Further, research has shown that jurors may have preferences for particular verdicts as the trial starts, which influence the verdicts they decide upon and how they evaluate evidence (Carlson & Russo, 2001). The availability of the *not proven* verdict may cause some jurors to favour this verdict option over *guilty* and *not guilty* verdicts (as shown in the research by Ormston et al., 2019), decreasing the chances of jurors favouring either the prosecution or defence evidence, thus leading to a lower frequency of *guilty* verdicts in the three-verdict system. Similar findings have been reported in more recent literature (see e.g. Curley et al., 2018, Lilley et al., 2022; Willmott et al., 2018).

Nevertheless, if the availability of the *not proven* verdict does decrease conviction rates, as suggested by some studies, then this may imply that the criminal justice system fails to provide justice for complainants. As there is a debate in the literature about the extent and significance of the effect of the *not proven* verdict on juror conviction rates, a quantitative meta-analysis on existing data sets from suitable studies seems timely and fitting.

To summarise, each of the studies highlighted above demonstrates that different verdict systems can influence how jurors reach their decisions (Curley et al., 2022). However, there is disagreement on the effect: for example, solely decrease *not guilty* verdicts (Curley et al., 2019; Hope et al., 2008; Smithson et al., 2007) or decrease both *guilty* and *not guilty* verdicts (Curley et al., 2022; Ormston et al., 2019). In other words, faced with an identical stimulus in the form of a mock trial, the propensity for jurors to convict may be affected by the number of verdicts available to them. However, these studies have different sample sizes, use different mock trials and present material in different ways. This makes it difficult to quantify and test the overall effect of verdict system in a conventional analysis, with some evidence to suggest that verdict choice is influenced by verdict systems (Ormston et al., 2019) and by crime type (Curley et al., 2022; Ling et al., 2021; Walker & Woody, 2011). Furthermore, since Smithson et al. (2007) published the first empirical paper on the *not proven* verdict, the body of research on this specific topic has grown. Still, there has been no attempt to combine findings across suitable studies in a quantitative meta-analysis to estimate the effect of verdict system. The results may inform the Scottish Government, which has announced plans to change the three-verdict system to a two-verdict system.

We can improve and clarify the effect of verdict system on juror decisions by considering not only juror verdicts from a single study or mock trial but juror verdicts from a range of studies that use the same experimental design. Despite the growing interest in meta-analyses, specifically in the context of juror decisions, most articles are either based on systematic literature reviews (e.g. Eatley et al., 2018, on the Crime Scene Investigation (CSI) effect; Hudspith et al., 2023, on rape myths) and scoping reviews (e.g. Leverick, 2020, on rape myths). Fewer studies have collated effect size measures across studies to provide improved inferential statistics (e.g. MacCoun & Kerr, 1988, on the leniency effect; Bystranowski et al., 2021, on the anchoring effect) but so far none has targeted the effect of verdict systems on conviction rates.

Here, we use the exact numbers of convictions and acquittals as reported in studies with matched mock trials to estimate the effect of verdict system. Introducing ‘verdict system’ as a fixed and ‘mock trials’ as a random factor in a logistic regression already establishes a meta-analysis (Cooper et al., 2019; Harbord & Whiting, 2009; Simmonds & Higgins, 2016) that can increase reliability and generalisability of estimated effects even if the number of studies is small (Yarkoni, 2020).

## Method

### Secondary data analyses

The selection of reports and studies for this meta-analysis followed PRISMA guidelines (Page et al., 2021). Full eligibility criteria and rationale are provided in Supplementary Materials (Table S1, https://osf.io/ybvpz). In short, we included quantitative studies that compare the number of juror convictions and acquittals in matched trials under the English/Anglo-American two-verdict and the Scottish three-verdict system. All relevant studies were identified through a comprehensive search of 11 databases in February 2022 using combinations of the keywords Juror bias* Juror research* Jury research* Mock Juror trial* Juror Simulation * Scottish verdict system* English verdict system* Scottish jury research* Cognitive bias in juries (Table S2 and S3 in Supplementary Materials).

The authors also carried out a ‘snowballing’ literature search to identify additional studies by searching the reference lists of publications in English. The studies and mock trials identified here are, to the best of our knowledge, the full extent of available data sets in this domain.

#### Inclusion/exclusion criteria

The inclusion criteria for studies and juror decisions were: (a) a random assignment of mock jurors to matched mock trials under two verdict systems, comparable to the English/Anglo-American two-verdict and the Scottish three-verdict system, (b) exact reporting of the number of mock juror decisions in terms of convictions and acquittals. Any studies or juror decisions that violated one of these criteria were disregarded from the meta-analysis.

Only reports in English were included. In the first main searches, 4,385 records were identified (see flow diagram in Figure S1, Supplementary Materials). After removal of duplicates and ineligible reports, 127 records were screened, and 103 of these could be excluded. The remaining 24 full texts were assessed, and a further 19 reports were excluded: 13 did not utilise verdict systems comparable to the English/Anglo-American two-verdict and the Scottish three-verdict system, and six did not report mock juror verdicts in terms of convictions and acquittals.

A total of 10 studies and mock trials from five reports were identified that used matched stimulus material in a between-subjects design. In each of the studies the same mock trial was presented to jurors under different verdict systems using a transcript, audio recording or full mock-trial video recording. Details of the mock trials are summarised under Materials and in Table 1 together with the number of convictions (*guilty*) and acquittals (*not guilty*; *not proven*) as reported for mock jurors randomly assigned to an English/Anglo-American two-verdict and the Scottish three-verdict system.

**Table 1.** Mock trials for mock jurors.

Mock trial	Design	Presentation	Crime	2-Verdict		3-Verdict	
				Convictions	Acquittals	Convictions	Acquittals
Smithson et al., 2007	B/W	Transcript	Homicide	9	43	5	47
Smithson et al., 2007	B/W	Transcript	Death by negligence	26	26	33	19
Smithson et al., 2007	B	Audio clip	Homicide	6	18	2	22
Hope et al., 2008	B	Transcript	Sexual assault^a^	27	17	31	29
Hope et al., 2008	B	Transcript	Physical assault	24	45	16	57
Ormston et al., 2019	B	Mock trial video	Rape	95	119	75	140
Ormston et al., 2019	B	Mock trial video	Physical assault	69	147	43	175
Curley et al., 2021	B/W	Vignette	Homicide	27	37	14	50
Curley et al., 2021	B/W	Vignette	Homicide	15	49	12	52
Curley et al., 2022	B	Mock trial video	Homicide	46	32	28	51

Note: Mock jurors were assigned to the English/Anglo-American two-verdict and the Scottish three-verdict system, where each mock juror was assigned to only one verdict system in each mock trial but made decisions in two different mock trials. B = between-subjects design, B/W = mixed between/within-subjects design; convictions = guilty; acquittals = not guilty, not proven. The odds ratios of each mock trial are not shown but correspond to the estimates presented in the forest plot of Figure 2.

^a^ This sexual assault trial may be better classified as a rape trial due to the description of the vignettes in Myers et al. (2003).

### Materials

In each of the 10 studies, participants were randomly assigned to different verdict systems but sometimes participants served as jurors in more than a single mock trial or gave more than one verdict. After following the evidence presented in the mock trial, each randomly assigned juror convicted or acquitted the accused or defendant using the two-verdict or the three-verdict system. Subsequent decisions by the same juror in a given trial were discounted to avoid carry-over effects.

In Mock Trials 1 and 2 (Smithson et al., 2007, Study 1) a total of *N* = 104 participants read a transcribed scenario of a criminal trial (homicide) and of a civil trial (death by negligence) and gave a verdict under a two-verdict as well as a three-verdict system in a mixed design with the order of mock trials and verdict systems counterbalanced across participants (order/verdict system – between subjects; mock trial – within subjects). We only considered the first and ignored the second verdict of each juror in a trial so that each participant gave a verdict under a single verdict system only. We treated the first verdict in each trial as independent observations although the same participant made juror decisions in each of the two mock trials.

In Mock Trial 3 (Smithson et al., 2007, Study 2) a total of *N* = 72 participants were assigned to three different groups. Two groups of 24 participants were assigned to an English/Anglo-American two-verdict system and the Scottish three-verdict system following a homicide trial. A further 24 mock jurors were assigned to a fictional verdict system and were therefore disregarded from the analyses. A third study (Smithson et al., 2007, Study 3) with a total *N* = 96 participants was disregarded for the same reason.

In Mock Trial 4 (Hope et al., 2008, Study 1) a total of *N* = 104 participants were assigned to the two-verdict system and three-verdict system in a between-subjects design passing their verdicts in a sexual assault trial. The description of the trial (Myers et al., 2003) suggests that according to the Sexual Offences Act (Scotland) 2009 the criminal case would be classified as a rape rather than sexual assault.^1^

In Mock Trial 5 (Hope et al., 2008, Study 2) a total of *N* = 142 participants were assigned to a two-verdict and three-verdict system in a physical assault trial. The juror verdicts in Study 2 were accumulated across three trial versions that featured weak, moderate and strong evidence.

In Mock Trial 6 (Ormston et al., 2019) a total of *N* = 429 and in Mock Trial 7 a total of *N* = 434 participants watched a video of proceedings in a rape and a physical assault trial, respectively. After watching the video they provided individual juror verdicts before (and after) deliberating in a jury. We only used juror verdicts before deliberation and disregarded 106 participants from both mock trials because they were not clearly assigned to one of the experimental conditions in a counterbalanced design. We treated juror decisions before deliberation as independent of jury size (12, 15) and voting rule (majority, unanimity) because both factors had no effect on these juror verdicts.

In Mock Trials 8 and 9 (Curley et al., 2019, 2021) two transcripts of different court trials (Vignettes 1 and 2) were presented to the same *N* = 128 participants in a mixed design. The order of vignettes and verdict systems was counterbalanced across participants. In the analysis of Curley et al. (2019) the juror verdicts were treated as independent observations by only using the verdicts from the first vignette presented to each participant. In an alternative analysis, Curley et al. (2021) used the verdicts from both vignettes.

In Mock Trial 10 (Curley et al., 2022) a total of *N* = 227 participants watched a video recording of a re-enacted court case on physical assault. In a between-subjects design three groups of participants decided as mock jurors under different verdict systems (English, Scottish, Experimental). We disregarded the verdicts of the 70 jurors who were assigned to the experimental verdict system that featured only *proven* and *not proven* as verdict options.

## Results

The data and analysis code are available at https://osf.io/ybvpz. Data were analysed using R, Version 4.0.0 (R Core Team, 2020), R-package *metafor* Version 2.4–0 (Viechtbauer, 2010). Since this is a secondary data analysis of previously published data no ethical approval or pre-registration is required.

We were interested in whether verdict system (Scottish, Anglo-American) in a between-participants design significantly affects conviction rates when combining data of 10 mock trials and different studies. Table 2 features the total number of convictions (*guilty*) and acquittals (*not guilty*; *not proven*) under the Scottish three-verdict and English two-verdict systems of all mock jurors pooled across trials.

**Table 2.** Number and percentage of convictions and acquittals for the Scottish three-verdict and Anglo-American two-verdict system.

Juror decision verdict system	Conviction		Acquittal		Total	
	*N*	%	*N*	%	*N*	%
3-Verdict	259	15	642	36	901	51
2-Verdict	344	19	533	30	877	49
Total	603	34	1175	66	1778	100

Note: Convictions = guilty; acquittals = not guilty, not proven. Juror verdicts are pooled across mock trials.

A Fisher’s exact test as well as Pearson’s chi-squared test with Yates’ continuity correction (Prescott, 2019) indicates that the odds ratio (*OR*) significantly deviates from 1 (*p* < .00001), suggesting that verdict system (three verdicts, two verdicts) and juror decisions (conviction, acquittal) are not independent. The *OR* estimated by maximum likelihood equals 0.625, 95% confidence interval, CI [0.51, 0.77]. This estimate uses pooled data and suggests that the odds of being convicted by jurors under the Scottish three-verdict system are 37.5% lower than those under the Anglo-American two-verdict system.

This odds ratio can be computed from the entries in Table 1. The odds for a conviction under the Scottish three-verdict system P(Conviction|Three Verdicts)/(1 − P(Conviction|Three Verdicts)) = (259:901)/(642:901) = 0.403 are divided by the odds for a conviction under the English verdict system P(Conviction|Two Verdicts)/(1 − P(Conviction|Two Verdicts)) = (344:877)/(533:877) = 0.645, resulting in an odds ratio *OR* = 0.403/0.645 = 0.625.

An alternative measure, based on the ratio of probabilities rather than odds, is the relative risk or risk ratio (*RR*). The numbers in Table 1 indicate a conviction rate of 344:877 or 39.2% under the two-verdict system and a lower conviction rate of 259:901 or 28.7% under the three-verdict system. The risk ratio between the verdict systems is *RR* = 0.287/0.392 = 0.732. According to this measure, jurors under the Scottish three-verdict system are 26.8% less likely to convict than jurors under the Anglo-American two-verdict system. An even more intuitive measure provides the risk difference *RD* = 0.392 – 0.287 = −0.105. This indicates a 10.5% reduction of the likelihood to convict when comparing the Scottish to the Anglo-American verdict system.

However, these results are based on pooled data and do not take into account different odds ratios and sample sizes of mock trials. We therefore conducted a logistic regression with random effects using the observed number of convictions and acquittals from each mock trial and verdict system. This method works well for small and stratified samples and leads to more precise estimates and statistical inference (Cooper et al., 2019). More conventional meta-analyses rely on effect sizes in less controlled studies with more complex designs and therefore require a larger number of studies (e.g. Milkman et al., 2021).

### Logistic regression with random effects

For the main analysis each individual verdict of a mock juror was categorised as a conviction or an acquittal, and the binary data served as the dependent variable. Studies with mock trials (labelled 1–10) are treated as a random effect. Introducing mock trials as a random effect is appropriate because they vary unsystematically across studies.

This approach makes weak assumptions about the underlying distributions and variance estimation but should give more robust and accurate results because it can accommodate extreme values, as well as varying and unbalanced sample sizes (Cooper et al., 2019; Harbord & Whiting, 2009; Simmonds & Higgins, 2016). In the following, we report the results of a logistic regression model with random effects, using restricted maximum likelihood estimation (REML), that is specifically adapted for meta-analyses and is implemented in the function rma ( ) of the R-package *metafor* (Viechtbauer, 2010).

By taking into account the different number of convictions and acquittals for each verdict system across the 10 mock trials, the estimated log odds for verdict system is −0.52, 95% CI [−0.77, −0.27]. This value is statistically significant (*z* = −4.10, *SE* = 0.13, *p <* .0001), and the corresponding odds ratio is *OR* = 0.59, 95% CI [0.46, 0.76]. This means that for all mock trials the odds of a conviction are reduced under the Scottish three-verdict system by a factor of 0.593, amounting to a change in odds by 40.7% compared to the Anglo-American two-verdict system. Note that the *OR* estimate is slightly lower and has a narrower confidence interval than that from Fisher’s exact test reported above (*OR* = 0.63, 95% CI [0.51, 0.77]).

**Figure 1.**
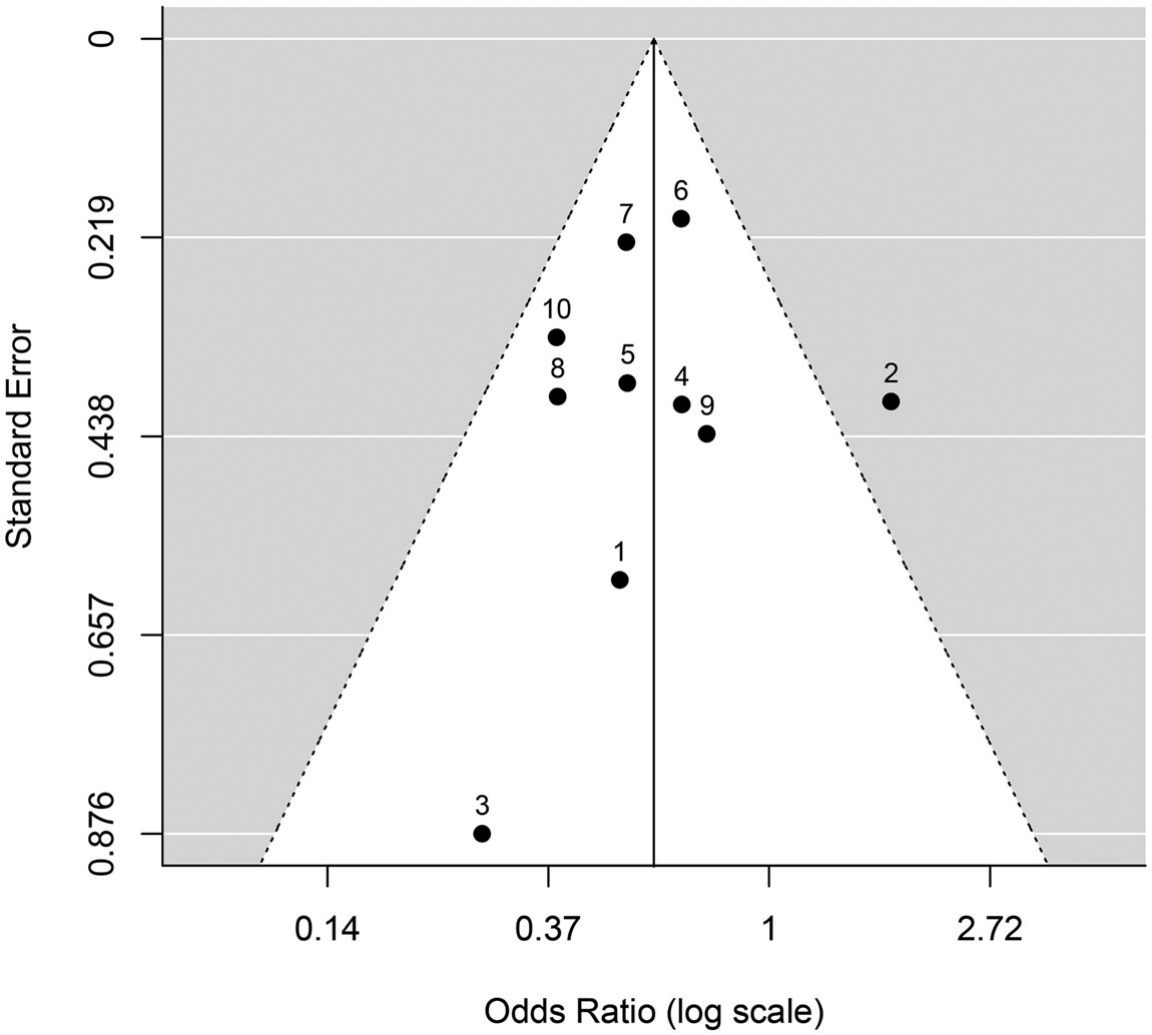
Funnel plot of odds ratios (log scale) centred on the odds ratio estimate 0.59 (vertical line) with standard errors (*SE*) of estimates on the *y*-axis. The number above each black data point refers to the corresponding mock trial.

In the funnel plot of Figure 1 each data point represents a mock trial with the standard error plotted against the odds ratio. The symmetric appearance of data points with a single outlier (Mock Trial 2) suggests no selection bias, and Kendall’s rank test for funnel plot asymmetry is not significant (*τ* = .02, *p* = 1.0). A test of heterogeneity is also not significant, Cochran’s *Q*(9)= 12.28, *p* = .198, *I*^2^ = 22.2%. The single outlier to the right of the funnel denotes the only ‘civil trial’ (Smithson et al., 2007, Study 2). In this mock trial a city council rather than a person was sued for death by negligence, which may explain the increased odds ratio. Excluding the data of Trial 2 had only a small effect on the results (*OR* = 0.55; 95% CI [0.44, 0.68]). The data point for Mock Trial 3 (Smithson et al., 2007, Study 2) at the bottom of the plot has the lowest sample size (*N* = 48) and the largest standard error among the 10 mock trials.

**Figure 2.**
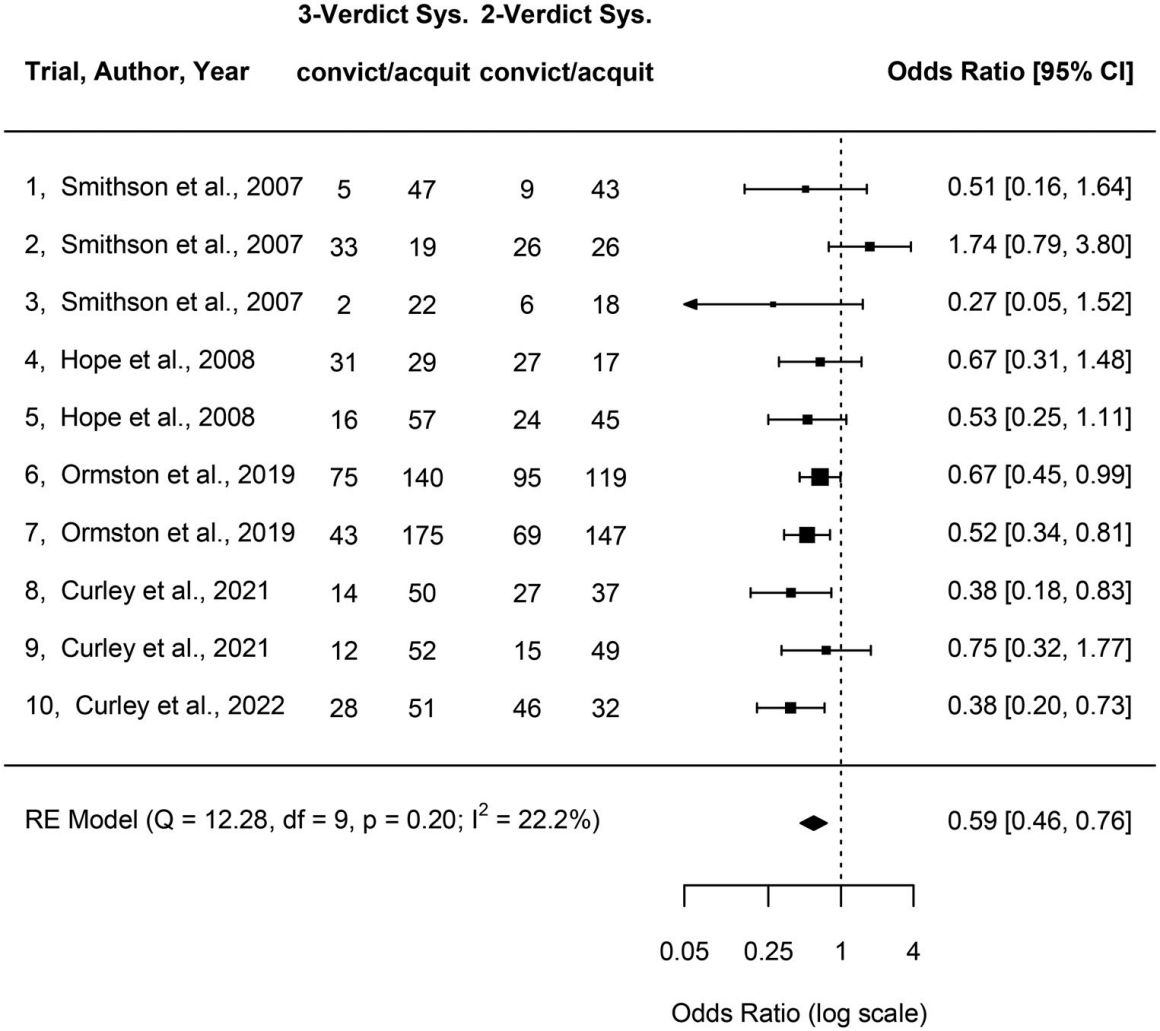
Forest plot of estimated odds ratios for 10 studies and mock trials based on a logistic model with random effects (Viechtbauer, 2010). CI = confidence interval. An odds ratio below 1.0 indicates a lower number of convictions for the 3-verdict system than for the 2-verdict system (see text for details).

In Figure 2 the results are summarised in a forest plot. The size of each black square corresponds to the sample size in the corresponding mock trial. The horizontal position of the squares indicates the estimated odds ratio (on a log scale), and the horizontal whiskers describe the 95% confidence intervals. Three out of 10 mock trials (Trials 7, 8 and 10) suggest odds ratios that are significantly lower than 1.0, whereas the estimates for the other mock trials tend to be more uncertain and therefore ambiguous. Only Trial 2 suggests an odds ratio higher than 1.0. The polygon or diamond at the bottom of the plot describes the summary estimate. The centre of the polygon corresponds to the point estimate of *OR* = 0.59, and the left and right edges indicate the 95% CI [0.46, 0.76]. Thus, the summary estimate is significantly lower than 1.0.

In an ancillary analysis each individual verdict of a mock juror was categorised into *not guilty* and *other*, with the latter category consisting of *guilty* and *not proven* verdicts for the three-verdict system. The resulting binary variable informs about changes in the number of *not guilty* verdicts when *not proven* is available or not. As before, verdict system (three verdicts, two verdicts) is entered as a fixed effect whereas trial (1 to 10) is treated as a random effect.

By taking into account the number of *not guilty* and *other* verdicts for each verdict system across the 10 mock trials, the estimated log odds for verdict system is −2.13, 95% CI [−2.82, −1.45]. This estimate are also statistically significant (*z* = −6.14, *SE* = 0.35, *p <* .0001). The corresponding odds ratio of *OR* = 0.12, 95% CI [0.06, 0.23], suggests a reduction of the odds for a *not guilty* verdict by a factor of 0.12 or 88% when comparing the three-verdict system to the two-verdict system.

## Discussion

In Scotland, jurors in criminal trials have three verdicts available to them (*guilty*, *not guilty* and *not proven*), whereas in England and Wales, and other common law systems such as the United States of America and Australia, there are only two verdicts (*guilty* and *not guilty*). The issue of whether juror verdicts might be affected by the number of verdict options available to them has been of some interest recently, in terms of both policy making and academic research. It is next to impossible to establish the true impact that different verdict systems might have on conviction rates in Scotland and England, for example, by simply comparing the reported conviction rates of trials in the two countries. The reason for this is that in addition to the different verdict systems, the Scottish and English legal systems have many other dissimilarities, in terms of jury size, majority rules for the jury and the way in which criminal offences are defined and admitted to court. For example, Scotland has a rule requiring corroboration of evidence, meaning that cases cannot proceed unless there are two sources of evidence of every ‘crucial fact’ (the identity of the perpetrator and the key offence elements). England does not have such a rule. This is likely to affect the number and type of cases that make it to trial in the first place, meaning that simply comparing actual conviction rates between countries would be misleading. For the same reason it would be problematic to include studies in the present meta-analysis that do not randomly assign jurors to different verdict systems and therefore have non-matching mock trials.

In order to investigate the effect of the three-verdict system compared to the two-verdict system it is therefore necessary to employ controlled experiments in which all features of the trial are held constant save for the verdict system. Such experiments typically use mock trials as a stimulus for jurors to reach a verdict, and a sufficiently large number of these experimental studies are now available to evaluate the results across studies and mock trials. In the present paper, we performed a quantitative meta-analysis on several experimental studies and found that despite considerable variability across studies and mock trials in terms of sample size, stimulus material, type of crime and conviction rates the results are quite unambiguous: there is a statistically significant effect towards lower conviction rates under the Scottish three-verdict system than under an Anglo-American two-verdict system. We estimate that under the Scottish compared to the Anglo-American verdict system the odds of a conviction are reduced by a factor of 0.593 (40.7%).

An equivalent analysis on *risks* or probabilities suggests a factor of 0.73, with the risk for a conviction reduced by 27% under the Scottish verdict system. The risk difference (*RD*) for convictions between the two verdict systems approaches *RD* = 11%.

This summary estimate appears to be the first of its kind in the domain of juror decisions and establishes a highly significant effect of verdict system on the odds of convictions or *guilty* verdicts. This is accompanied by significantly lower odds for *not guilty* verdicts than for *guilty* and *not proven* verdicts, which are reduced by a factor of 0.12 (88%) when comparing the Scottish three-verdict system with the Anglo-American two-verdict system.

Taken together, the results demonstrate a robust and significant effect of verdict system on juror decisions across mock trials, ranging from death by negligence to physical assault, rape and homicide. Introducing mock trials as a random factor in a logistic regression improves estimation because it helps to explain variability in the data (Cooper et al., 2019). This increases robustness and generalisability of overall estimates and statistical power (Yarkoni, 2020).

There are a number of possible psychological explanations for the effect of verdict system. One general explanation comes from the field of bounded rationality, which states that decision making of an individual is shaped by both their cognition and the decision environment (Simon, 1956). Bounded rationality is a concept that suggests that decision-making capabilities of individuals are limited by the information they can collect, their cognitive limitations of information processing and the finite amount of time they have to reach a decision. It suggests that people make decisions based on ‘satisficing’ rather than strict maximising principles.

Applied to decision making by jurors, bounded rationality suggests that the decision-making process has internal constraints (e.g. processing limits, both in terms of information storage and processing speed), as well as external constraints (e.g. how complex the external information is and/or how costly it is to search for information). Additionally, time constraints may prevent jurors from fully considering and integrating all the evidence and arguments before rendering a decision.

As a result of these constraints, jurors may employ heuristics or mental shortcuts to make decisions. For example, in extreme cases jurors may rely on stereotypes, emotions or prior beliefs to form a verdict. These factors can influence the decision-making process and may lead to decisions that are not considered fully rational (Curley et al., 2021). Similarly, the process during jury deliberation may also be constrained by the fact that jurors often try to reach a consensus where some jurors may be more persuasive and influential than others (Clark et al., 2007). Group dynamics and social pressure may also affect the final jury verdict. Furthermore, as the verdict system is changed from a two-verdict system to a three-verdict system, the decision environment changes, potentially introducing more complexity and ambiguity to the decision process and therefore increasing the use of heuristics, which ultimately affects conviction rates (Curley et al., 2022).

Another reason for why the availability of the *not proven* verdict relates to a lower conviction rate may be due to attention being on ‘proof’ rather than ‘truth’. In the three-verdict system, there are two acquittal verdicts, one with a focus on proof, which may raise scepticism of jurors towards the prosecution’s evidence, thus tilting the scales of justice in favour of the defence. The removal of the *not proven* verdict may also stop jurors using it as a convenient compromise. Due to the verdict falling in the middle of *guilty* and *not guilty*, its absence forces jurors to ‘pick a side’, with some jurors choosing *not guilty* and some choosing *guilty*, causing an increase in both when compared to a three-verdict system (Hope et al., 2008; Smithson et al., 2007). Although the odds are larger for *not guilty* versus *other* than for *guilty* versus *other* (acquittals), it still runs contrary to the legal perspective that if jurors fail to reach a decision beyond the threshold of reasonable doubt in a three-verdict system, then they should choose a *not guilty* verdict in a two-verdict system.

In their seminal paper, Carlson and Russo (2001) showed that jurors tend to settle on verdicts early on in a trial, which causes them to favour evidence that supports their initial verdict and distort or disregard evidence that does not confirm their initial verdict. The availability of the *not proven* verdict implies that jurors are more likely to favour the ‘middling verdict’ from early on in a trial. This means that they are less likely to be biased to the arguments of either the prosecution or the defence team, decreasing their chances of reaching a *guilty* and *not guilty* verdict and thus leading to a lower number of convictions in a three-verdict system.

The statistically significant effect of verdict system on conviction rates indicates an obvious decision bias in jurors. Although trials and legal consequences remain the same, introducing a second label for acquittal reduces conviction rates of individual jurors. Therefore, juror decisions must be exposed to some form of cognitive bias. More specifically, under both verdict systems jurors are asked to assign different labels to what is essentially a binary choice: to convict or acquit. Under an Anglo-American verdict system *guilty* and *not guilty* verdicts map directly onto conviction and acquittal. Under the Scottish verdict system *guilty* also maps onto conviction whereas *not guilty* and *not proven* both map onto acquittal. The third option *not proven* simply offers an alternative label to acquit the defendant/accused but a rational decision maker should not be affected by this third option (Chalmers et al., 2022; Hope et al., 2008).

In preference tasks and consumer behaviour (Huber et al., 1982; Kahneman & Tversky, 1984) introducing a third option or ‘decoy’ can drive a decision maker who is indifferent about two options toward one of the options. A successful decoy facilitates ‘asymmetric dominance’, which requires attributes on two dimensions with indifferent information between two of the options but a clear dominance of one option over the decoy. In the context of juror decisions, the two dimensions may be described as the ‘presumption of innocence’ of the defendant and ‘proof beyond reasonable doubt’ against the defendant, not unlike the two concepts of ‘truth’ and ‘proof’. If a juror is indifferent between a *guilty* and *not guilty* verdict, then the introduction of *not proven* as a third verdict option may shift jurors’ decision towards an acquittal because *not proven* reminds jurors that without evidence beyond reasonable doubt the defendant should be acquitted even if they are believed to be guilty.

The aspiration that a verdict in a court case is objective and rational or unbiased and unprejudiced appeals to everyone whereas the presence of bias or prejudice that may affect jurors’ decisions seems wrong and harmful (Sherrod, 2019). However, the presumption of ‘innocence until proven guilty’ and providing ‘proof beyond reasonable doubt’ may trigger different predispositions in jurors’ decision making. Therefore, attempting to eradicate cognitive bias is quite challenging and may conflict with instructions given to jurors. Verification of a verdict is also extremely difficult to achieve because this requires not only identification and validation of previously unknown facts in a court case (e.g. guilt or innocence of the accused, validity of existing evidence and witnesses, new evidence), but also application of the same normative rules and standards (e.g. criminal proceedings, juror selection, interpreting laws and societal norms), and even prediction of future events (e.g. consequences of a verdict on victim(s), the accused, and society in general).

### Limitations

There are a number of limitations in the present analyses that need to be acknowledged. An exhaustive search identified a total of only 10 studies and mock trials with varying numbers of jurors and odds ratios for conviction as well as ecological validity.

Ecological validity refers to the extent to which a mock trial reflects the features of a real criminal trial. For example, does the mock trial use: (a) accurate legal directions, (b) realistic evidence presented by trained actors and (c) a representative (e.g. community) sample of mock jurors rather than relying on student participants (Bornstein, 1999)? The higher the ecological validity of the experiment, the more likely it is that the findings can be replicated in real criminal trials. Some of the studies that are included here had relatively low ecological validity and, for example, used trial transcripts or audio vignettes. This is not surprising because creating a realistic audio-visual stimulus of proceedings in court is expensive and time consuming. Nevertheless, the largest data sets included in the meta-analysis – that of Ormston et al. (2019) and of Curley et al. (2022) – had the highest ecological validity. Ormston et al. used hour-long trial videos, scripted in collaboration with legal professionals and performed by actors in a real courtroom. Curley et al. used a 53-minute-long video featuring legal professionals and a real judge, also in a real courtroom, with actors playing the roles of the witnesses. In both studies jurors were given the same legal directions as they would be given in a real trial and were drawn from the local population to reflect the demographic make-up of real juries.

Another possible limitation would be the presence of publication bias (van Aert et al., 2019). The meta-analysis is based on the number of convictions and acquittals from 1778 jurors from 10 studies published in journal articles between 2007 and 2022. Publication bias may have prevented the publication of null effects or contradicting results. However, we are not aware – through searches of theses, conference abstracts and personal communications with researchers in the field – of unpublished studies in this domain, and the funnel plot in Figure 1 does not suggest an asymmetry.

Another limitation of the present analysis may be the degree to which each of the studies varied in relation to crime type (death by negligence, rape, homicide and physical assault). Research has shown that verdicts can vary for different crime types (Ormston et al., 2019). Increasing the number of mock trials would have enabled us to introduce a moderator variable such as crime type. However, we only identified 10 studies in the literature that systematically manipulated verdict system for matching mock trials. Despite this limitation, the present meta-analysis informs the Scottish Government about the consequences of abolishing the *not proven* verdict. Combining the findings from a small but controlled body of studies on the *not proven* verdict makes it possible to establish a robust statistically significant effect of verdict system on juror decisions.

Finally, the strong effect of verdict system on juror decisions does not necessarily translate into an equivalent effect on jury verdicts. In real criminal trials, verdicts are delivered by juries after deliberation, usually in a group of 12 jurors (or, in Scotland, a group of 15 jurors). In most jurisdictions, verdicts have to be (near-)unanimous, although in Scotland juries can deliver majority verdicts (Chalmers et al., 2020). This means that individual juror verdicts may change over the course of the deliberations in order to facilitate agreement, questioning whether jurors’ pre-deliberation verdicts reliably predict jury verdicts. In Ormston et al. (2019) jury deliberations were included in the research design, and jury verdicts were recorded for a total of 64 juries in a counterbalanced design (2 verdict systems, 2 trial types, 2 jury sizes and 2 majority rules). In only one instance (simple majority required, three-verdict system, 15-person jury) mock jurors were significantly more likely to give a *guilty* verdict post-deliberation than pre-deliberation. This suggests that deliberation may have a leniency effect on juror decisions. The exclusion of deliberations in jury research may decrease the ecological validity of the studies in the current analysis. However, only one jury study that involved jurors deliberating collectively in a jury was available, which is a more general issue (Curley & Peddie, in press; Diamond, 1997). Future research into the *not proven* verdict, and other relevant factors, should include jury deliberations and jury verdicts.

### Implications

In 2023, the Scottish Government introduced legislation to abolish the *not proven* verdict, which, at the time of writing, was being consulted on. If this legislation gains parliamentary approval and becomes law, this would bring Scotland in line with the vast majority of legal systems worldwide in having only two verdicts that can be returned after a criminal trial – *guilty* and *not guilty*. The results of the present meta-analysis suggest that this may lead to a significant increase in convictions. This is, of course, on the assumption that the results of the experimental studies can be translated into the context of real-world trials. As we noted above, this is not an assumption that can be made, given the differences between the experimental studies and real criminal cases and trials (Curley & Peddie, in press; Diamond, 1997). Nevertheless, what can be concluded with a reasonable degree of certainty is that if the abolition of the *not proven* verdict does have an effect on conviction rates, and without any further changes being made to the legal system (juror size, majority rule), it will be in the direction of an increased number of convictions across most trial types.

This is a change that would be welcomed by groups who argue that justice is not being secured for those who have experienced sexual assault. In recent years, much of the criticism of the *not proven* verdict has come from Rape Crisis Scotland, who have advocated for its abolition (Rape Crisis Scotland, 2021). Rape and sexual offence cases have a far lower conviction rate than trials for other serious offences, and the *not proven* verdict is used disproportionately in such cases (Chalmers et al., 2021a). Research has suggested that jurors sometimes use the *not proven* verdict as a compromise verdict to bring deliberations to a close rather than resolve disagreements or uncertainty (Chalmers et al., 2022). This is especially concerning in rape cases and sexual offence cases, where it is well documented that jurors exhibit other biases such a reliance on rape myths (Chalmers et al., 2021b; Leverick, 2020).

There is also the danger that an increase in conviction rates might be an increase in wrongful convictions – the conviction of the factually innocent. This seems unlikely to occur in the arena of sexual offences, where conviction rates are low (see Chalmers et al., 2021b), but could be a genuine problem in other contexts – for example, when the evidence is primarily based on eyewitness identification or a confession (Chalmers et al., 2022). The *not proven* verdict has traditionally been seen as one of the major protections against wrongful conviction in Scotland, and removing it without putting other safeguards in place may well increase the risk of wrongful convictions.

All of these points are necessarily speculative. It is near impossible to identify the ‘correct’ rate of convictions that the criminal justice system should be returning. Criminal trials are contested versions of reality, and even the parties directly involved might not recall or know everything that happened in the context of an alleged crime. As changes are made, however, policy makers should be mindful that other protections may need to be implemented in order to reduce the risk of wrongful convictions. These may include changes to the majority rule for conviction (whereby a *guilty* verdict can be returned if only 8 of the 15 jurors favour *guilty*) or to the rules relating to particular types of evidence, such as confessions or eyewitness identification evidence.

## Conclusion

The highly significant effect of verdict system on conviction rates does not simply confirm earlier findings from single studies but establishes for the first time a reliable estimate and statistical test of the verdict effect across mock trials. Thus, the main result from the present meta-analysis is that, across studies and trial types, the Scottish three-verdict system reduces the odds of a conviction by 40.7% and the probability of a conviction by 10.5% compared to an Anglo-American two-verdict system.

The more fundamental question of whether abolishing the *not proven* verdict and the likely increase in convictions may benefit or harm defendants, victims and the wider society is far more difficult to address because this requires validation of verdicts in criminal legal trials as ‘correct’ or ‘true’. Such a validation is very difficult, if not impossible, because it relies on gathering new information, sometimes long after a first trial (e.g. through technological advances, confessions). Nevertheless, only such a validation would allow determining whether a change in conviction rates constitutes a successful adaptation of the legal system or not.

## Supplementary Material

Supplemental Material

## Footnotes

1 This is because the Sexual Offences (Scotland) Act 2009 defines rape as ‘penetration of the vagina, anus or mouth of another person by the penis without consent’. The trial clearly deals with penetration, and as all rape trials are a type of sexual assault trial but not all sexual assault trials are a type of rape trial, rape is a more legally accurate trial type to explain the vignettes used in the Myers et al (2003) and Hope et al (2008) studies.

## Supplemental data

Supplementary data and analysis code can be accessed at https://osf.io/ybvpz
