# Supplementary material for: The effect of verdict system on juror decisions: a quantitative meta-analysis
Source: Psychiatr Psychol Law. 2024 Jan 11;32(1):23–41. doi: 10.1080/13218719.2023.2272912 (PMC11774159; doi:10.1080/13218719.2023.2272912)
Supplement: Supplemental Material [file TPPL_A_2272912_SM1958.docx]

**Supplementary Information (https://osf.io/ybvpz)**

**Table S1. Eligibility criteria and rationale**

**From:** **The Effect of Verdict System on Juror Decisions: A Quantitative Meta-Analysis**

| Criterion | Rationale |
| --- | --- |
| A random assignment of mock jurors to matched mock trials with verdict systems, comparable to the English/Anglo-American two-verdict and the Scottish three-verdict system. | Several research groups have empirically investigated the effect that the *not proven* verdict, as a third option, has on juror decision making. However, a meta-analysis on conviction rates using matched trials under different verdict systems has not been conducted. |
| Detailed reporting of the number of mock juror decisions in terms of convictions and acquittals. | As there is a debate in the literature about the extent of the effect of the *not proven* verdict on juror conviction rates, a comprehensive quantitative analysis on existing data seems timely and fitting. |

# Table S2. Databases searched

**From:** **The Effect of Verdict System on Juror Decisions: A Quantitative Meta-Analysis**

| Database | Coverage |
| --- | --- |
| PsychInfo | 1980 to Present |
| PsychArticles | 1980 to Present |
| Pubmed | 1980 to Present |
| Applied Social Sciences Index & Abstracts | 1980 to Present |
| ProQuest | 1980 to Present |
| Heinonline | 1980 to Present |
| JSTOR Law | 1980 to Present |
| Nexis | 1980 to Present |
| Scopus | 1980 to Present |
| Psychology and Behavioural Sciences Collection | 1980 to Present |
| Web of Science | 1980 to Present |

# Table S3. String of search terms

**From:** **The Effect of Verdict System on Juror Decisions: A Quantitative Meta-Analysis**

| String of search terms |
| --- |
| *“Juror bias” or “Juror research” or “Jury research” or “Mock Juror trial” or “Juror Simulation” or “Scottish verdict system” or “ English verdict system” or “Scottish jury research” or “Cognitive bias in juries”*. |

**Figure S1. Flow diagram of search (Page et al., 2021)**

Records identified from databases:

PsychInfo (n = 220)

PsychArticles (n = 29)

Pubmed (n = 868)

Applied Social Sciences Index & Abstracts (n = 109)

Heinonline (n = 236)

JSTOR Law (n = 4)

Nexis (n = 350)

Scopus (n = 179)

Psychology and Behavioural Sciences Collection (n = 44)

Web of Science ( n = 249)

ProQuest (n = 2097)

Records removed *before screening*:

Duplicate records removed (n = 1494)

Records marked as ineligible by manual screening (n = 1352)

Records removed for other reasons (n = 1412)

**Identification**

Records screened

(n = 127)

Records excluded**

(n = 103)

Reports sought for retrieval

(n = 24)

Reports not retrieved

(n = 0)

**Screening**

Reports assessed for eligibility

(n = 24 )

Reports excluded:

Reason 1 (n = 13)

Reason 2 (n = 6)

Reports included in review

(n = 5)

**Included**
